# Bidirectional Cross-Linguistic Interference in Spatial Cognition: Behavioural Evidence from Chinese Learners of French

**DOI:** 10.3390/bs16030332

**Published:** 2026-02-27

**Authors:** Lin Xue, Zhong Chen, Zichun Xu, Yanru Zhang

**Affiliations:** 1 School of Foreign Languages and Literature, Shandong University, Jinan 250100, China; l.xue@sdu.edu.cn; 2 Department of Chinese Language and Literature, University of Macau, Macao, China; zhongchen@um.edu.mo; 3 Hopkins–Nanjing Center for Chinese and American Studies, Nanjing University, Nanjing 210093, China; zxu151@jh.edu

**Keywords:** spatial cognition, spatial prepositions, bidirectional transfer, French as a foreign language, Mandarin Chinese, behavioural task

## Abstract

This study investigates how cross-linguistic differences in spatial cognition affect Chinese learners’ acquisition of French in the conflict domain of page turning, which is encoded in opposite ways by French and Mandarin. Two hundred and sixty-one Chinese university students completed a video-based spatial task in both languages, comprising both comprehension and production components. The results revealed a marked asymmetry in spatial cognition between the first language (L1) and second language (L2): while learners consistently relied on stabilised Mandarin-based construals, their French responses remained strongly shaped by L1 frames of reference. We found no significant association between global French proficiency and success in the French spatial tasks, indicating that higher proficiency does not automatically entail conceptual restructuring in this domain. Meanwhile, a small to moderate negative correlation between French and Mandarin scores indicated a subtle L2-to-L1 influence, whereby adopting French-conventional spatial construals was accompanied by reduced alignment with Mandarin-conventional patterns. These findings contribute to research on bidirectional cross-linguistic influence in spatial cognition by documenting L2-to-L1 effects in late, classroom-based learners. They also point to the need for pedagogical approaches that explicitly target spatial conceptualisation—through contrastive reflection and embodied practice—rather than focusing solely on the formal properties of spatial expressions.

## 1. Introduction

Learning a second or foreign language involves more than acquiring new lexical items and grammatical forms. As Vygotsky (1986/2012) argued, language does not merely express thought; it also shapes it. The close relationship between language and cognition is particularly evident in the conceptualisation of space (Talmy, 1985; Levinson, 1996), a domain in which languages differ markedly in their structural, referential, and cultural patterns (Lakoff & Johnson, 1980; Boroditsky, 2001; Lin, 2022; Lum et al., 2022). In this study, we use the term ‘spatial cognition’ to refer to the mental representation and processing of spatial relations as mediated by language; that is, how spatial concepts are encoded, accessed, and recruited in linguistic use. As a result of cross-linguistic differences in spatial language, the same movement event may be interpreted in radically different ways across languages, depending on how spatial relations are encoded and construed.

Against this background of cross-linguistic variation in spatial cognition, when learners encounter a second language (L2) that implements a system of spatial reference distinct from that of their first language (L1), they face a dual challenge: mastering new linguistic forms while also restructuring their underlying mental representations of space. This tension is visible in processes of nativisation (Narcy-Combes et al., 2019)—the transfer of entrenched L1 patterns to the L2—and in the difficulty of ‘denativising’ these patterns, especially when learning starts relatively late. Yet most empirical work on spatial cognition in second language acquisition (SLA) has focused on English (L. Liu & Zhang, 2009; Ji, 2020; Z. Chen, 2021); much less attention has been paid to the specific effects of learning French, for example, for Chinese learners.

In this respect, Chinese learners of French as a foreign language (FLE) offer a particularly revealing case. Spatial expressions in French and in Mandarin differ substantially because they draw on distinct systems of reference (Levinson, 1996; Talmy, 2000). This is especially evident in the use of the basic localisation terms *avant* and *arrière*, which play a central role in structuring spatial cognition. In most contexts, the French *avant* roughly corresponds in meaning to the Mandarin *qian*, and *arrière* corresponds to *hou*, as illustrated below:

(1) *Tāmen de fángzi zài wǒmen de fángzi qiánmiàn.*
  ‘Their house is in front of ours’.
(2) *Háizimen zuò zài chē de hòupái.*
  ‘The children are sitting in the back seat of the car’.

However, a striking inversion occurs for certain specific activities, such as page turning. In French, *tourner la page vers l’avant* (lit. ‘turn the page forwards’) describes a movement that in Mandarin is encoded as *xiang hou fanye* (lit. ‘turn the page toward the back’), while *tourner la page vers l’arrière* (lit. ‘turn the page backwards’) corresponds to *xiang qian fanye* (lit. ‘turn the page toward the front’). In other words, *avant* aligns with *hou* and *arrière* with *qian* in this context, revealing an apparent contradiction in how the same movement is conceptualised in the two languages. This conceptual asymmetry raises a particular challenge for Chinese learners of French at both the linguistic level (i.e., selecting appropriate expressions) and the cognitive level (i.e., adjusting entrenched spatial representations).

Chinese learners represent a growing segment of the FLE population, both within formal education in China (with more than 100,000 learners reported in 2022; Ambassade de France en Chine, n.d.) and in Chinese communities in France, Canada, and other French-speaking countries and regions (Statistics Canada, n.d.; Rouhban, 2024). Despite this growth, however, Chinese learners of FLE remain largely under-represented in research on spatial cognition in FLE. This is unfortunate, as their linguistic profile could provide valuable insights for French language pedagogy and for supporting cognitive adaptation in multilingual settings.

In view of these gaps, the present study examines the interaction between French and Mandarin systems of spatial cognition among Chinese learners of FLE in a university context. Using an experimental design centred on a video-based page-turning task, we analyse learners’ performance in the comprehension and production of spatial expressions involving *avant* and *arrière* and *qian* and *hou* in both languages, to evaluate how conceptual divergences impact language learning. We then discuss the implications of our findings for FLE pedagogy, particularly for classroom instruction that addresses cross-linguistic differences in spatial conceptualisation and for supporting Chinese learners’ cognitive adaptation in multilingual environments. By linking language-mediated spatial cognition with both L1 and L2 performance in this underexplored linguistic pairing, the study also contributes to ongoing research on how language-specific conceptual patterns interact.

## 2. Literature Review

### 2.1. Spatial Cognition and Its Linguistic Encoding

Understanding how spatial cognition is structured and encoded in language is central to explaining cross-linguistic differences in spatial expression and their potential impact on L2 learning. Early discussions of the link between spatial cognition and language can be traced back to Whorf (1956), who, drawing on indigenous languages such as Hopi, argued that linguistic structures shape speakers’ habitual patterns of thought, including how spatial relations are conceptualised and organised. As Whorf famously put it, ‘we dissect nature along lines laid down by our native languages’ (Whorf, 1956, p. 213), suggesting that the categories through which people interpret spatial experience are not given directly by perception alone but partly shaped by the linguistic systems through which experience is organised. Fillmore’s (1975) theory of frame semantics further highlights the role of concepts such as agent, location and goal in the construal of spatial scenes, strengthening the connection between spatial cognition and language. Subsequent cross-linguistic work has provided empirical support for these theoretical claims. Bowerman (1996), for example, showed that children learning different languages become sensitive to different spatial distinctions depending on how these are lexicalised in their language, suggesting that linguistic categories guide attention to particular features of spatial scenes. Similarly, Pederson et al. (1998) demonstrated that speakers of languages favouring different spatial frames of reference adopt corresponding strategies in non-linguistic spatial memory tasks, indicating that habitual linguistic encoding is linked to broader cognitive orientations. These findings indicate that spatial language does not merely describe spatial cognition but helps to shape habitual patterns of spatial attention and reasoning.

To provide a more explicit cognitive–linguistic account of how spatial relations are conceptualised in language, Langacker (1987) introduced the trajector–landmark model, according to which spatial conceptualisation relies on the distinction between a focal entity (the trajector) and its spatial background (the landmark). In everyday language use, this model can be observed in expressions such as ‘the cup is on the table’, where the cup functions as the trajector and the table as the landmark. The same figure–ground alignment is evident in dynamic descriptions such as page turning, where either the page or the reader’s perspective may serve as the reference point. These examples illustrate how spatial meaning arises from asymmetries in attention and conceptual salience.

These insights were further systematised and extended in Levinson’s (1996, 2003) work on the linguistic encoding of space, which provides the main theoretical basis for our experimental design. Levinson distinguished three main frames of reference: an absolute frame, based on fixed coordinates such as cardinal directions; a relative frame, anchored in the observer’s viewpoint and expressed through terms such as ‘left’ and ‘right’; and an intrinsic frame, where spatial relations depend on the inherent properties of the reference object, such as its canonical front or back. Levinson (1996) further demonstrated that these linguistically preferred frames of reference are reflected in non-linguistic spatial cognition. For example, speakers of Tzeltal, a Mayan language that predominantly uses an absolute frame of reference based on uphill/downhill distinctions tied to the local topography, systematically relied on these geocentric coordinates when performing memory and reasoning tasks. After being rotated 180 degrees, they tended to reconstruct spatial arrays according to fixed environmental directions (e.g., ‘uphill’ vs. ‘downhill’) rather than according to their own left–right perspective. In contrast, speakers of languages favouring relative frames of reference typically reproduced the same arrays using viewer-centred coordinates. This cross-modal alignment between linguistic encoding and spatial reasoning suggests that habitual patterns of language use are systematically associated with preferred cognitive strategies for organising space.

A comparable contrast can be observed between Mandarin and French, the two languages examined in the present study. Mandarin speakers often favour an intrinsic frame in certain spatial contexts, where relations are defined by the characteristics of the objects or actions involved. French, in contrast, relies more heavily on relative frames based on the observer’s perspective or on relations between objects. This contrast helps explain why some actions, such as page turning, are described in opposite ways in French and Mandarin, and it suggests that speakers of the two languages may rely on different underlying spatial frames of reference when conceptualising the same physical event. From an intrinsic Mandarin perspective, turning a page ‘forward’ (*xiang hou fanye*) is determined by the movement of the page itself, whereas in French, *vers l’avant* reflects a relative perspective centred on the user. In our study, this divergence between intrinsic and relative frames is operationalised through a page-turning task in the two languages.

In parallel, Talmy (2000) proposed a typology that classifies languages according to how they encode motion events, distinguishing between verb-framed languages such as French, in which motion is typically encoded in the verb (e.g., *entrer dans la pièce*, *lit.: enter (in) the room*), satellite-framed languages such as English, where motion is expressed in satellites (e.g., *run into the room*), and mixed systems such as Mandarin Chinese, which combine both strategies. This typology underscores the flexibility of Mandarin in encoding spatial relations (Z. Chen, 2021), in contrast with the more tightly constrained syntactic patterns of French.

Taken together, the models of Levinson (1996, 2003), Langacker (1987) and Talmy (2000) offer complementary perspectives on the interface between language and spatial cognition. Levinson (1996, 2003) and Talmy (2000) emphasised the influence of language on the structuring of spatial thought, whereas Langacker (1987) adopted a more interactional view, according to which cognition and language shape each other. These studies converge on the idea that language not only represents a neutral medium for describing space but actively structures how space is perceived and conceptualised, while leaving open important questions about how pre-linguistic spatial cognition and linguistic systems influence each other over time. More recent work on embodied cognition has added a neurocognitive and developmental dimension to this picture. Gallese and Lakoff (2005) argued that conceptual knowledge, including action- and space-related meanings, is grounded in the sensory–motor system, suggesting that the comprehension of spatial language involves embodied simulation mechanisms. From a developmental perspective, Simms and Gentner (2019) demonstrated that children use spatial terms to structure their understanding of complex spatial relations, such as orientation and position, and that knowledge of specific spatial words predicts success in non-verbal spatial reasoning tasks.

### 2.2. Spatial Cognition in Second and Foreign Language Learning

Given its strong contextual and cultural dependence, the linguistic encoding of spatial relations has attracted growing attention in research on SLA and foreign language learning. Cross-linguistic studies have shown that languages differ markedly in how they structure spatial relations and frames of reference (e.g., Bowerman & Choi, 2001; Coventry & Guijarro-Fuentes, 2008). The impact of the L1 on L2 learning is frequently described in terms of conceptual transfer (Odlin, 1989; Slobin, 1996; Jarvis & Pavlenko, 2008; Alonso et al., 2016; Alhammad, 2023; Phuc et al., 2023), whereby entrenched L1-based construals of space shape how learners interpret and encode spatial relations in an additional language. From this perspective, speakers of different languages are seen as construing spatial relations in systematically different ways, and these entrenched construals shape how learners encode space in the L2. At the same time, SLA research in other conceptual domains shows that L2-based routines can also become established in speakers’ cognition. For example, Athanasopoulos (2009), Athanasopoulos and Bylund (2013) and Bylund and Athanasopoulos (2014) demonstrated that with increasing L2 experience, bilinguals’ performance in non-verbal tasks involving colour categorisation and temporal judgements gradually shifted towards patterns that are typical of the L2, rather than simply preserving L1-based preferences. These findings suggest that conceptual distinctions acquired in an additional language can, under certain conditions, reshape underlying representational habits.

In line with this view, several authors have argued that the L1 of L2 users is not an isolated, closed system but permeable to the influence of other languages in the speaker’s repertoire (Langacker, 1987; Cook, 2003; Jarvis, 2007; Jarvis & Pavlenko, 2008). While the influence of the L1 on the L2 has been extensively documented, the reverse effect—how L2 learning may feed back into cognition and use in the L1—has received far less systematic attention (Pavlenko, 2011). Studies of L2-to-L1 influence, although not all focused exclusively on spatial cognition, have shown that acquiring another language can modify patterns of conceptualisation in the first language by fostering hybrid ‘thinking-for-speaking’ modes that draw elements from both languages (Ameel et al., 2009; Park & Ziegler, 2014) and by modulating how events are mentally represented and remembered in the L1 (Filipović, 2022). More direct evidence of L2-to-L1 effects in event construal comes from research on motion expression, which has highlighted the role of factors such as age of onset, length of residence and L2 proficiency in shaping bilinguals’ L1 performance (Bylund, 2009; Brown & Gullberg, 2011). Taken together, these studies indicate that L2 experience can, under certain conditions, feed back into L1 cognition, even if spatial cognition is not always the sole focus of analysis. They therefore support a dynamic view of the relationship between language and spatial cognition, in which conceptual patterns—including those structuring spatial experience—remain open to cross-linguistic influence across the lifespan.

### 2.3. Research Gaps and Research Questions

Although research has provided valuable insights into the interaction between language and cognition in L2 learning, two important gaps remain. First, most studies have examined domains in which the L1 and L2 profile events differently (e.g., in terms of endpoint salience or path segmentation), rather than cases in which the two languages encode the same spatial movement in opposed ways. Domains in which two languages offer conflicting construals of the same event—known as ‘conflict domains’—provide a particularly sensitive testbed for L2-to-L1 influence. In such domains, maintaining two incompatible mappings (e.g., *avant* vs. *qian*/*hou* in page turning) requires continuous cognitive control, and any L2-driven restructuring in the L1 should become especially visible. Second, the vast majority of experimental research on spatial cognition in SLA has focused on English or on interactions between European languages, including work with Chinese learners (Li & Zhang, 2019; Ji, 2020; Z. Chen, 2021). The specific case of French, which is typologically distant from Mandarin, has been largely neglected.

To help address these gaps, the present study investigates the interaction between Mandarin and French systems of spatial cognition among Chinese university students learning FLE, using page turning as a case study. It addresses the following questions:

1. To what extent is Chinese learners’ mastery of French spatial expressions related to their proficiency in French?
2. Is there a relationship between learners’ performance in spatial expressions in Mandarin and in French, and if so, what does this reveal about cross-linguistic conceptual transfer?

## 3. Materials and Methods

### 3.1. Participants

The study drew on a sample of 261 learners of French enrolled in five Chinese universities located in three key provinces in eastern and central China. The institutions included two top-tier national research universities and three specialised universities with strong foreign language programmes. This diversity allowed us to cover a range of academic contexts and strengthened the geographical and institutional representativeness of the sample.

To minimise potential bias linked to institutional profiles, the selection of universities also took into account the 2024 national ranking of French programmes (ShanghaiRanking, 2024). Two institutions were ranked at level A, one at level B+, and one at level B; one had not been formally evaluated and was therefore considered “unranked” for the purposes of this study.

All of the participants were undergraduate or master’s students, and most of them were between 18 and 24 years old, which corresponds to the typical age range of university-level FLE learners in China. With regard to their proficiency in French, 128 participants had never taken a formal proficiency test, while the others had passed at least one of the following standardised exams: TFS-4, TFS-8, TCF/TEF and DELF/DALF^1^. TFS-4 and TFS-8 are specialist French tests specifically designed to assess the competence of French majors in mainland China. For participants who had taken formal proficiency tests, scores from DELF/DALF, TCF/TEF, and TFS were mapped onto CEFR levels according to the official equivalence tables provided by the respective testing organisations. The various formal tests reported by the participants were aligned with CEFR levels. DELF/DALF, TCF, and TEF are officially calibrated against CEFR. The Chinese TFS, widely used in university French programmes, is interpreted as follows (Bohec, 2015; Yu, 2021): TFS-4 corresponds approximately to B1; passing TFS-8 corresponds to B2; and a high distinction in TFS-8 is treated as C1.

For students without an official test certificate, self-reported CEFR levels were used. In the Chinese university context, formal proficiency tests in French are typically taken from the third year onwards^2^. As a result, most first- and second-year students in the present sample did not yet hold an official certificate and were classified based on self-assessment. To assess whether self-reported CEFR levels displayed a progression pattern consistent with expected institutional training trajectories, we compared their distribution across study stages (Table 1). Students in Years 1–2 were predominantly located in the A1–A2 range, whereas higher CEFR levels were increasingly represented among students in Years 3–7, who had taken formal tests. The overall distribution of self-assessed levels followed the same broad progression pattern observed in institutional advancement, providing indirect support for the internal plausibility of the proficiency grouping.

Based on their test results and self-assessed CEFR level, the students were divided into two proficiency groups: Group A (*n* = 110), comprising learners at elementary level (A1–A2), and Group B (*n* = 151), comprising learners at higher levels (B1–C1). The broader grouping of upper-level learners was motivated by the uneven distribution of proficiency levels in the sample. In the Chinese university context, French is typically learned from an absolute-beginner starting point, and only a relatively small proportion of students reach the C1 level or above by the end of their undergraduate studies. This makes finer-grained categorisation of advanced levels statistically less robust. Table 2 presents the distribution of the participants by university and proficiency group.

In addition to the learner sample, a control group of 37 native speakers of French was recruited to provide a baseline for performance in the French spatial tasks. These participants were university students in France with no knowledge of Mandarin. They completed the same French comprehension and production tasks as the learner group, using identical video stimuli and procedures. The inclusion of this control group made it possible to assess whether learner performance approached native-like patterns and to evaluate the relative difficulty of the French items.

### 3.2. Materials

Data were collected through an online video-based questionnaire consisting of two parts. The first part gathered background information on the participants’ gender, age, first and additional languages, experience of taking French proficiency exams (TFS-4, TFS-8, TCF/TEF, DELF/DALF), and self-assessed CEFR (Common European Framework of Reference for Languages) level (A1–C2). These data were used to assign students to the two proficiency groups and provided a basis for subsequent comparative analyses.

The second part comprised four experimental questions designed to probe the spatial representations mobilised in a Mandarin–French bilingual context through a familiar and culturally neutral action: turning a page. Drawing on Levinson’s (2003) work on frames of reference and Z. Chen’s (2021) research on Mandarin–English spatial encoding, the tasks were constructed to capture both comprehension and production—that is, the interpretation of an observed action and the selection of an appropriate action to perform.

Each task type was implemented in both languages, with a passive version (interpreting a gesture) and an active version (choosing a gesture to perform) in Mandarin and in French, yielding four conditions in total. Each condition was presented via a short standardised video clip followed by a multiple-choice question. Using homogeneous visual stimuli limited contextual variation and ensured the comparability of responses across languages. To minimise experimenter effects, all instructions were pre-recorded and administered uniformly.

**Figure 1:**
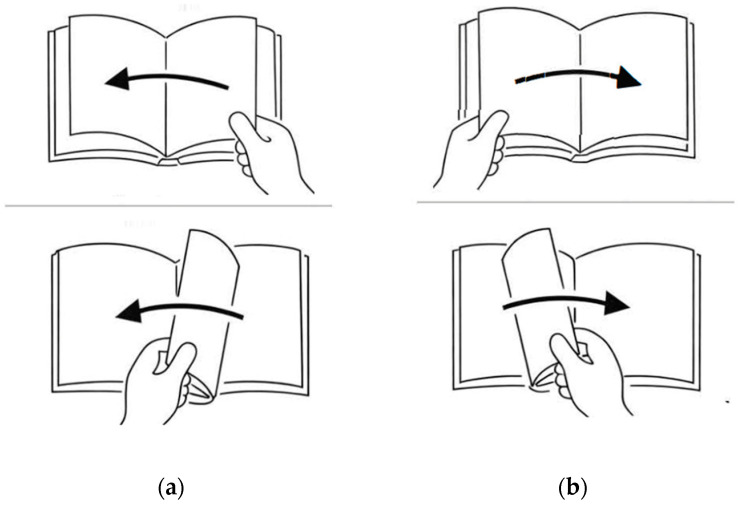
(**a**) Schematic representation of the page-turning movement shown in Video S2 (‘backward’ turn); (**b**) Schematic representation of the page-turning movement shown in Video S1 (‘forward’ turn).

The original video stimuli are represented schematically in two line drawings (Figure 1), which illustrate the movements of forward and backward page turning, respectively. Table 3 summarises the four experimental questions, indicating the language, task type and corresponding video clip(s). For the native French control group, only the French tasks (Q3–Q4) were administered, as these items established a native baseline for the target spatial construals.

### 3.3. Procedure

The questionnaire was first reviewed by experts in FLE and spatial cognition and piloted with a small group of students to check clarity and reliability. Minor adjustments were made following the pilot phase. The final version was then administered online over a two-week period. Invitations to participate were distributed through the five participating universities.

To complement the quantitative data collection described above, the study adopted a mixed-methods design. In this design, questionnaire data were complemented by semi-structured interviews. The interviews were conducted with five students, selected from the top-performing learners in each university on the basis of their academic records. The interviews were designed to deepen the interpretation of the quantitative results by exploring how the participants reflected on the tasks, reasoned about their responses, experienced the differences between Mandarin and French spatial expressions, and reconsidered their intuitive responses after being shown the correct answers.

The research protocol was reviewed and approved by the Ethics Committee of the authors’ institution. The study followed standard ethical guidelines for research involving human subjects. The study procedures were explained, and the participants gave informed consent electronically before taking part. All data were anonymised at the point of collection and used solely for research purposes.

### 3.4. Data Analysis

Three main variables were defined for the quantitative analyses. The first was French proficiency group, coded as Group A (value = 1, elementary level) and Group B (value = 2, higher level), based on the combination of self-assessment and standardised test results.

The other two variables captured performance in the spatial cognition tasks. The Mandarin score reflected the number of correct responses to the two Mandarin questions, on a scale from 0 to 2 (0 = no correct answers, 1 = one correct answer, 2 = two correct answers). The French score was defined analogously for the two French questions, using the same 3-point scale.

The data were analysed with SPSS 19.0 in three steps. First, descriptive statistics were computed to summarise the participants’ characteristics and overall performance in the spatial tasks. Second, non-parametric tests were used to examine group differences. Mann–Whitney U tests assessed differences between the two proficiency groups, and Kruskal–Wallis tests were used to explore variations in Mandarin scores as a function of French scores. Third, Spearman rank-order correlations were calculated to examine the relationships between French proficiency group, Mandarin scores and French scores.

The quantitative results were interpreted with reference to conventional statistical criteria to evaluate the study’s hypotheses and identify relevant links between the linguistic and cognitive variables under investigation. The interview data were used to provide additional contextual insight and enrich the interpretation of the quantitative patterns.

## 4. Results

### 4.1. Descriptive Statistics

Descriptive analyses revealed a clear contrast between performance in the French and Mandarin tasks.

**Figure 2:**
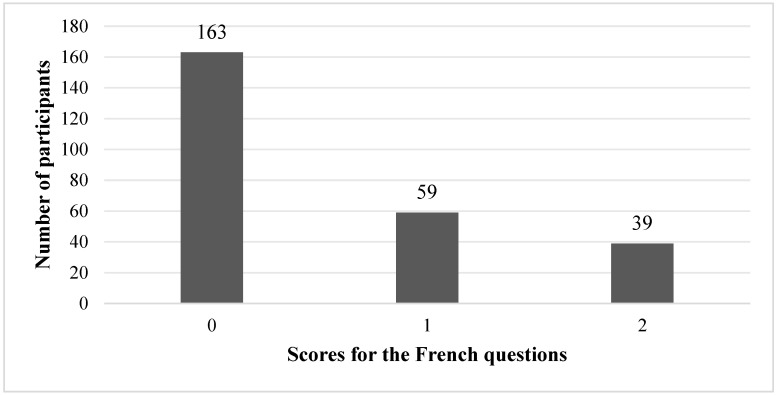
Bar chart of the number of participants obtaining each score for the French questions.

For the French questions, most of the participants obtained low scores: 163 learners scored 0, 59 scored 1, and only 39 achieved the maximum score of 2 (Figure 2). This distribution indicates that a large proportion of learners did not systematically select the French-conventional construals in the spatial tasks.

**Figure 3:**
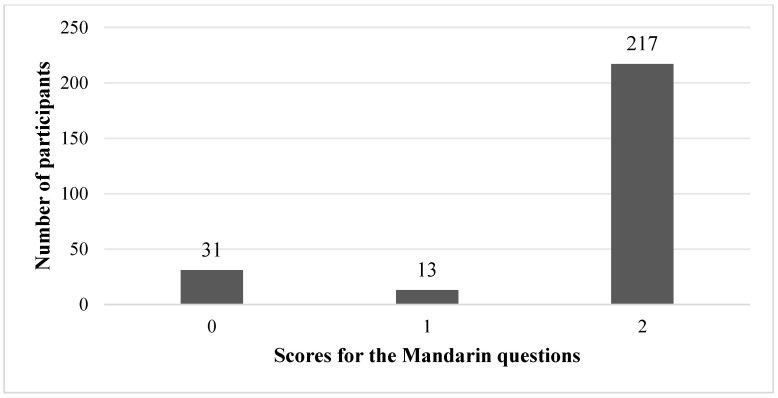
Bar chart of the number of participants obtaining each score for the Mandarin questions.

Performance in the Mandarin questions was considerably higher. As shown in Figure 3, 217 participants obtained the maximum score of 2. Overall, these descriptive patterns point to a marked asymmetry between L1 and L2 performance: while Mandarin spatial construals appeared to be highly stabilised, the corresponding French patterns were much less firmly established in this learner population.

To assess performance in the two language tasks in greater detail, responses were examined at the item level. In the Mandarin tasks, the accuracy rates for the two items were both high and consistent (comprehension: 85.4%; production: 85.8%). This convergence suggests that the L1-congruent construal of page turning was highly stable and easily retrieved in both the interpretive and action choice scenarios. In the French tasks, performance was significantly lower, with a slight asymmetry between the task types. The accuracy rate for the comprehension item was only 21.8%, while that for the production item was 30.7%. Although both values indicate substantial divergence from the French-conventional construals, the higher success rate for production may reflect different task requirements: choice of video may rely more on procedural decision-making strategies, while selecting a direction label may more directly invite comparison with L1-based conceptualisations.

Overall, the item-level results confirm that the cross-language contrast observed in the aggregated scores was not driven by a single anomalous item but reflected a consistent pattern across both comprehension and production tasks in each language.

**Figure 4:**
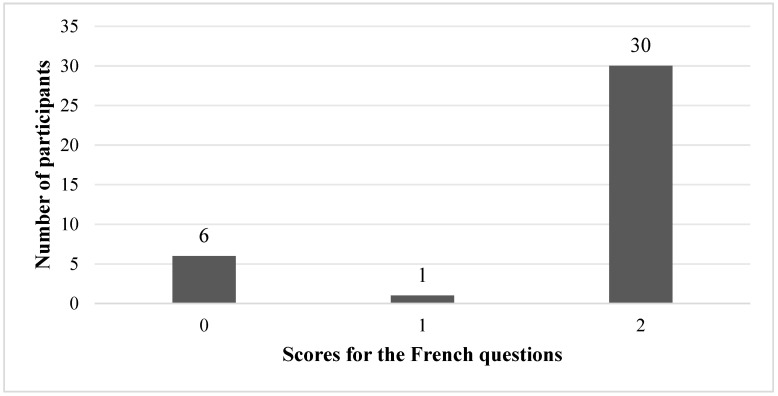
Bar chart of the number of native French speakers obtaining each score for the French questions.

To contextualise these findings, the performance of a native French control group (*n* = 37) was examined descriptively (see Figure 4). As expected, the native speakers showed a near-ceiling performance on the French items, with the majority obtaining the maximum score. This pattern suggests that the lower performance observed in the learner group is unlikely to be explained solely by task ambiguity and is consistent with an interpretation centred on difficulty in adopting French-conventional spatial construals.

### 4.2. Proficiency-Group Comparisons

To assess whether French proficiency was associated with performance in the spatial tasks, two Mann–Whitney U tests were conducted to compare the French and Mandarin scores of Group A (elementary level, A1–A2) and Group B (higher level, B1–C1). For the French questions, the Mann–Whitney U test yielded a *p*-value of 0.056, with a small effect size (r = 0.12); for the Mandarin questions, the *p*-value was 0.915 and the effect size was negligible (r = 0.01). As both *p*-values exceed the conventional α = 0.05 threshold, these tests did not indicate statistically reliable differences between the two proficiency groups in terms of either French or Mandarin performance in the spatial tasks.

To provide a complementary and group-based perspective on the relationship between L2 and L1 task performance, a Kruskal–Wallis test was then conducted. The participants were grouped into three categories according to their French scores (0, 1, or 2), and their Mandarin scores were compared across these groups. The results, summarised in Table 4, showed a significant effect of French score group on Mandarin performance (χ^2^ = 42.693, *p* < 0.001, ε^2^ = 0.16); however, this analysis represented a categorical view of the same underlying association, which was later quantified using Spearman correlation analysis. Mean rank values indicated that learners with higher French scores tended to obtain lower scores in Mandarin for the spatial tasks.

### 4.3. Spearman Correlations

To directly examine the strength and direction of the associations among French proficiency level, French task performance, and Mandarin task performance, Spearman rank-order correlations were calculated.

As shown in Table 5, neither the correlation between proficiency group and French score (r_s_ = −0.119, *p* = 0.055) nor that between proficiency group and Mandarin score (r_s_ = −0.007, *p* = 0.915) reached statistical significance. In contrast, a small to moderate negative correlation was observed between the French and Mandarin scores (r_s_ = −0.338, *p* < 0.001). This pattern indicates that higher performance in the French spatial tasks was associated with lower performance in the equivalent Mandarin tasks, and vice versa.

### 4.4. Interview Findings

The semi-structured interviews provided qualitative insights that aided interpretation of these quantitative patterns. Three of the five interviewed students reported that during their FLE education, they had never been explicitly exposed to the idea that spatial cognition might differ across languages. One participant noted: *‘We were taught directional vocabulary*, *but nobody ever told us that the way of thinking about space could be different in French’.*

The remaining two interviewees indicated that they had not noticed any divergence between Mandarin and French spatial systems before taking part in the experiment. One commented: *‘I thought “vers l’avant” meant the same thing in every language. I had never thought that it could depend on culture or on the writing system’.*

A lack of explicit awareness also emerged with respect to the L1. One participant expressed surprise at having answered one of the Mandarin questions incorrectly and reported questioning her own intuition in her first language: *‘I even made a mistake for the Mandarin question. I still don’t really understand why. Maybe I never really thought about what “front” or “back” actually mean’.*

Four out of five students reported a tendency to transfer their habitual Mandarin-based spatial representations to French without conscious adjustment. After being shown the correct answers, all expressed surprise at the discrepancy between their intuition and French conventions. One learner summarised this as follows: ‘*I realised I was answering in Mandarin*, *even when the question was in French. It’s unsettling*, *but also very interesting*’. These reflections suggest that raising learners’ awareness of cross-linguistic differences in spatial conceptualisation may foster more reflective learning processes. Encouraging learners to question their intuitive spatial interpretations could therefore support deeper metacognitive engagement in FLE learning.

## 5. Discussion

### 5.1. Limited Link Between Linguistic Proficiency and Spatial Cognition

The quantitative results revealed a pronounced asymmetry between L1 and L2 performance in the spatial tasks. Only 14.9% of the participants achieved the maximum score for the French questions (score = 2; Figure 2), whereas 83.1% obtained the maximum score for the Mandarin questions (Figure 3). Neither the non-parametric group comparisons nor the correlation analyses revealed a significant association between French proficiency level and performance in the French or Mandarin spatial tasks. In other words, learners with higher global proficiency in French did not systematically select the French-conventional construals of the page-turning event. Item-level analyses further suggest that the overall asymmetry was not driven by a single anomalous item or by random responding. Mandarin accuracy was consistently high across both comprehension and production, whereas French performance remained low in both conditions, with only a modest advantage for production. This consistency across task types supports the interpretation that the difficulty is likely to reflect cross-linguistic conceptual conflict rather than being a task-specific artefact. This pattern departed from the intuitive expectation—and the finding of some previous work on event construal (e.g., Bylund, 2009; Brown & Gullberg, 2011)—that increasing L2 proficiency facilitates the adoption of L2-typical conceptualisations. Instead, it suggests a persistent reliance on L1-based frames of reference, even among students at the B1–C1 level.

The observed persistence is consistent with research showing that spatial expressions, particularly spatial prepositions, often constitute a long-term area of difficulty in L2 learning, because they are anchored in deeply entrenched conceptual schemas rather than in simple form–meaning pairings (Tyler et al., 2011; De Knop, 2020; Boieblan, 2023). Our learners’ interview comments reinforced this interpretation: several explicitly stated that they had ‘never thought about’ the possibility that French might encode spatial relations differently from Mandarin and realised, after the task, that they were ‘answering in Mandarin even when the question was in French’. Such remarks point to a systematic gap between the formal acquisition of lexical and grammatical resources and the appropriation of the underlying spatial schemas that organise their use.

A second lens through which to interpret these findings is the notion of *negative conceptual transfer* (De Knop, 2020; Gu, 2024), which posits that the greater the typological distance between the L1 and L2 in a given conceptual domain, the more difficult it is for learners to reorganise entrenched L1 schemas without targeted conceptual instruction. In the present case, Mandarin directional verbs and French prepositions encode page-turning events in directly opposed ways, creating precisely the kind of conflict domain in which L1 habits are likely to dominate unless they are explicitly problematised in the classroom. Recent neuroimaging work has further suggested that early bilingual experience is associated with more efficient cortico-cerebellar connectivity supporting the coordination of linguistic and cognitive processes (Gracia-Tabuenca et al., 2024). In contrast, our participants, who typically began French at the age of 18, were late L2 learners, for whom large-scale neural reorganisation may be more limited. Taken together, these considerations support the view that incremental gains in grammatical or lexical proficiency are not, by themselves, sufficient to trigger the restructuring of deeply rooted spatial schemas: cognitive realignment requires explicit, conceptually oriented pedagogical intervention.

From an embodied-cognition perspective, this resistance to change is unsurprising. Based on previous studies and the qualitative data obtained in this study, the Chinese higher education system relies primarily on cognitively and textually oriented approaches, which often overlook the bodily and sensory dimensions of learning (Cuet, 2020; Xu, 2023; Zhang, 2023), while linguistic cognition is deeply rooted in multimodal experiences—visual, sensory and kinaesthetic—acquired during early language use (Barsalou, 2008; Ahlberg et al., 2018). When L2 learning requires a radical reconfiguration of these embodied schemas (as in the present case, where French spatial prepositions differ markedly from Mandarin directional verbs and are associated with opposite gestures), adult learners face a well-documented form of cognitive inertia (L. Liu & Zhang, 2009; Yang & Wen, 2017). The slight advantage observed for the French production task over the comprehension task may tentatively be viewed in this light. Although both tasks relied on visual stimuli, the production condition required the participants to select an action direction, which may have engaged more procedural or action-oriented decision processes, potentially more compatible with emerging L2 routines. The comprehension task, being more interpretation-focused, may have been more susceptible to interference from entrenched L1-based conceptualisations. In such circumstances, cognitive realignment requires explicit conceptual work on how space is construed, rather than only additional practice with linguistic forms.

### 5.2. Associations Between L1 and L2 Performance in a Spatial Conflict Domain

Beyond the absence of any association between overall French proficiency and performance in the spatial tasks, the present study revealed a small to moderate negative correlation between scores in French and in Mandarin. Learners who were more likely to select the French-conventional construals for page turning tended to be less consistent in selecting the Mandarin-conventional construals in the equivalent L1 tasks. This pattern is compatible with an interpretation in terms of L2-to-L1 conceptual influence: experience with the L2 does not simply add a new set of spatial schemas but may also, in specific domains, be associated with less consistent selection of L1-conventional construals. In this respect, our results resonate with Brown and Gullberg’s (2011) finding that L2 experience, even for late classroom learners with intermediate proficiency, was associated with measurable differences in L1 event construal. At the same time, our results nuance the view that adult L1 systems remain globally stable and are affected mainly at the level of lexical access (Köpke & Schmid, 2004; Bylund, 2009): even without dramatic ‘attrition’ in the traditional sense, we observed patterns of performance variation in narrowly defined conceptual domains.

A key feature of the present study is that it targeted a conflict domain, the highly familiar action of turning a page, for which French and Mandarin offer directly opposed mappings. This contrasts with most previous studies of cross-linguistic influence in motion expression, which have compared how events are profiled (Bylund, 2009; Filipović, 2022) rather than considering cases of outright reversals. Domains in which two languages prescribe incompatible construals for the same action are theoretically likely to be particularly relevant contexts for examining possible L2–L1 interactions, because maintaining both mappings requires continuous conflict monitoring and control at the conceptual level. This is in line with psycholinguistic work showing that bilingual speakers rarely operate in a ‘single-language mode’: even when one language is clearly dominant and the other is acquired later, the non-target language tends to be co-activated, and language use involves ongoing cross-language competition and control (Thierry & Wu, 2007; Kroll et al., 2015). In such a dynamic system, adopting new spatial routines in French may involve additional control demands when learners perform a formally similar task in Mandarin, particularly in a conflict domain where the two languages encode ‘forward’ and ‘backward’ in opposite ways.

Cognitive neuroscience research has offered further support for this view at a more mechanistic level. Studies using structural and functional imaging have shown that L2 acquisition can lead to reorganisation in cortical and subcortical regions implicated in higher-order cognition, including networks relevant to spatial processing and cognitive control (Pliatsikas, 2020; Pliatsikas et al., 2020). Complementary evidence from functional near-infrared spectroscopy suggests that advanced L2 users recruit additional neural resources to inhibit the non-target language during complex bilingual tasks (Cong et al., 2021). While these studies did not deal specifically with page turning, they converged on the idea that managing competing linguistic systems carries a cognitive cost that can, under certain conditions, be associated with differences in how events are represented during task performance.

From this perspective, L2 learning cannot always be viewed simply as an additive enrichment of the linguistic repertoire. Even in late classroom learners in a non-immersion context, it may be associated with variability in L1 task performance in domains where the two languages impose conflicting spatial mappings. This pattern becomes especially visible in such conflict configurations, as illustrated here by the French–Mandarin page-turning contrast. This type of configuration could be further tested across additional language pairings to shed light on universal versus language-specific patterns of spatial cognition.

## 6. Conclusions

This study examined how learning French interacts with spatial cognition in a conflict domain where French and Mandarin encode the same everyday action—turning a page—in directly opposed ways. Using a video-based task with 261 Chinese university students, we found a strong asymmetry between L1 and L2 performance and, crucially, no reliable link between global French proficiency and success in the French spatial tasks. The observed small to moderate negative correlation between French and Mandarin scores suggests that adopting French-conventional construals may be accompanied by reduced alignment with Mandarin-conventional patterns. Taken together with the interview data, these findings add to current debates on bidirectional cross-linguistic influence by showing that even for late classroom learners in a non-immersion context, L2 experience can modulate L1-based spatial routines in subtle but systematic ways.

These observations have direct pedagogical implications. They argue for a pedagogy that is both reflective and embodied. On the reflective side, learners need structured opportunities to compare spatial descriptions across languages, analyse apparently incorrect but intuitive answers, discuss contradictory gestures and verbalise the strategies they use when translating or interpreting spatial expressions. Such activities can foster metacognitive awareness of how L1 frames of reference shape perception and expression and help learners recognise and manage negative conceptual transfer (De Knop, 2020; Jessner et al., 2021; Gu, 2024; Y. Liu, 2024).

On the embodied side, our findings suggest that FLE instruction in spatial prepositions should move beyond purely textual and decontextualised exercises to deliberately build in bodily experience. In practical terms, this means designing tasks in which students *physically* enact spatial meanings—for instance, walking to different locations in the classroom, manipulating objects or actually turning pages *vers l’avant/vers l’arrière*—while verbalising what they are doing in French and comparing it with Mandarin. Such activities are consistent with evidence that bodily engagement can enhance understanding and retention of linguistic concepts and support the reorganisation of underlying schemas (Asher, 1977; Barsalou, 2008; Mavilidi et al., 2015; Niemeier, 2017; Skulmowski & Rey, 2018; Schmidt et al., 2019; Suñer & Roche, 2021; Gamba Kresh, 2022). In conflict domains such as page turning, teachers could also combine explicit work on the geometrical and functional properties of prepositions with guided movement, as shown to be beneficial for spatial expression (Boieblan, 2023), and systematically link diagrams and gestures to learners’ own embodied and sociocultural experience (Della Putta & Suñer, 2023). Recent tools such as generative artificial intelligence and virtual reality can be used to extend these principles by creating multisensory simulations of authentic situations, allowing learners to *experience* and discuss French–Mandarin conflicts in spatial construal rather than only reading about them (Y. Chen & Zhang, 2023; Xu, 2025).

Several limitations invite further research. First, although the sample size was sufficient for the statistical analyses conducted, larger and more diverse participant groups would increase statistical power and allow for finer-grained comparisons across proficiency levels and learner profiles. In addition, the study did not systematically take into account participants’ knowledge of other foreign languages, particularly English, which is widely learned in the Chinese context and shares the same page-turning logic with French. Future work could explore trilingual constellations to disentangle the combined effects of multiple L2s on spatial cognition (Jessner et al., 2021).

Second, the study adopted a cross-sectional design, which captures spatial cognition at a single point in time. Longitudinal research would be valuable to trace how learners’ spatial representations evolve with continued exposure to French and Mandarin. In addition, the present participants were relatively homogeneous in age, educational background, and learning context. Expanding the paradigm to learners from more varied sociocultural and educational backgrounds would help researchers assess the generalisability of the observed patterns.

The qualitative component, while designed to complement the quantitative results, involved a limited number of interviewees; future studies could include a broader range of learner voices to further explore individual differences in metacognitive awareness and experiential factors. Finally, the experimental design deliberately focused on a single, tightly controlled conflict-domain action (page turning) to isolate differences in conceptual mapping across languages. Extending the approach to a wider range of spatial actions and comparing task formats with varying degrees of visual or embodied support, as well as different stimulus-presentation modes and task-order configurations (e.g., randomisation), would help determine how methodological factors interact with cross-linguistic spatial representations in both research and instructional contexts.

## Figures and Tables

**Table 1:** Distribution of self-assessed CEFR levels by study stages.

University Year	A1–A2	B1–B2	C1–C2
Years 1–2	75.8%	24.2%	—
Years 3–7	9.8%	71.4%	18.8%
Total	42.1%	48.3%	9.6%

**Table 2:** Participants’ backgrounds.

University	National Ranking of French Programme (2024)	Group A Participants (A1–A2)	Group B Participants (B1–C1)	Total
University A	Level A	23	40	63
University B	Level A	24	37	61
University C	Level B+	19	32	51
University D	Level B	24	28	52
University E	Not ranked	20	14	34
Total	—	110	151	261

**Table 3:** Overview of the experimental questions.

Question	Language	Task Type	Stimulus/Stimuli
Q1	Mandarin	Comprehension (choice of direction)	Video S1
Q2	Mandarin	Production (choice of video)	Video S1 vs. Video S2
Q3	French	Comprehension (choice of direction)	Video S2
Q4	French	Production (choice of video)	Video S1 vs. Video S2

**Table 4:** Kruskal–Wallis test: Mandarin scores as a function of French score group.

French Question Score Group	N	Mean Rank	χ^2^	df	*p*-Value
0	163	141.61	42.693	2	<0.001
1	59	132.47			
2	39	84.44			

**Table 5:** Spearman correlations between proficiency group, Mandarin score and French score.

Variables	Coefficient (r_s_)	*p*-Value
French proficiency level and score for French questions	−0.119	0.055
French proficiency level and score for Mandarin questions	−0.007	0.915
Score for French questions and score for Mandarin questions	−0.338	<0.001

## Data Availability

The datasets generated and analysed during the current study are not publicly available because the participants did not provide explicit consent for open data sharing beyond the current project. Subject to approval by the relevant institutional ethics authorities and with the participants’ consent, anonymised data may be made available from the corresponding author upon reasonable request.

## Notes

1 TFS-4 and TFS-8 (*Test de français standardisé*) are specialist proficiency tests for French majors in mainland China, whereas TCF/TEF (*Test de connaissance du français*/*Test d’évaluation de français*) and DELF/DALF (*Diplôme d’études en langue française*/*Diplôme approfondi de langue française*) are international standardised French proficiency exams administered under the authority of the French Ministry of National Education.

2 TFS-4 is first taken near the end of the second academic year (typically in May), with the results released several months later, during Year 3 (in around September).

## Supplementary Materials

The following supporting information can be downloaded at: https://youtu.be/38ri1sYJZ-s?si=ZeWqstVqgzR7PP-m (Video S1) (accessed on 20 January 2026); https://youtu.be/RVCufrGb-1s?si=Ng6j4AaoxiONwu87 (Video S2) (accessed on 20 January 2026).
